# Do young children, like young adults, remember animates better than inanimates?

**DOI:** 10.3389/fpsyg.2023.1141540

**Published:** 2023-05-10

**Authors:** Aurélia Bugaiska, Patrick Bonin, Arnaud Witt

**Affiliations:** LEAD-CNRS UMR 5022, University of Burgundy, Dijon, France

**Keywords:** animacy effect, children, recollection, episodic memory, Remember/Know

## Abstract

It has repeatedly been shown in adults that animates are remembered better than inanimates. According to the adaptive view of human memory this is due to the fact that animates are generally more important for survival than inanimates. Animacy enhances not only the quantity but also the quality of remembering. The effect is primarily driven by recollection. Virtually all studies have been conducted in adults, and we believe that the investigation of animacy effects in children is also highly relevant. The present study therefore tested the animacy effect on recollection in young (6–7 years, *M* = 6.6 years) and older children (10–12 years, *M* = 10.83 years) using the Remember/Know paradigm. As found in adults, an animacy effect on memory was found, but only in older children, and specifically in the “remember” responses, suggesting, once again, its episodic nature.

## Introduction

Adaptive memory was first described by Nairne et al. (2008), Nairne (2010, 2015), Nairne and Pandeirada (2016). The authors postulated that human memory has evolved as a result of pressures faced by our ancestors in the distant past. According to this theory, memory is enhanced when the information is relevant to fitness and survival. A number of studies have provided evidence supporting this view (see Nairne et al., 2017a for a comprehensive review), including animacy effects in memory (e.g., Nairne et al., 2013; Bonin et al., 2014; for a review: Nairne et al., 2017b).

The animacy effect concerns the observation that animate entities (e.g., *snake*, *cow*, and *woman*) are remembered better than inanimate entities (e.g., *mountain*, *bottle*, and *car*). Importantly, it was by adopting an evolutionary lens to the study of (episodic) memory that Nairne et al. (2013) first demonstrated the importance of the mnemonic dimension of animacy. Because animates have a stronger fitness value than inanimates (i.e., they can be predators, prey or potential sexual partners), they predicted and then empirically demonstrated that animates have a memory advantage over inanimates. Animacy effects in memory have been found by different research teams world-wide, first in the United States (e.g., VanArsdall et al., 2013), followed by researchers in Europe [e.g., France (Bonin et al., 2014), Germany (Meinhardt et al., 2018)], and also in China (e.g., Li et al., 2016). The memory benefit of animacy has been found with different types of stimuli: words (Nairne et al., 2013; Bonin et al., 2014), non-words linked to animate vs. inanimate properties (VanArsdall et al., 2013), and pictures (Bonin et al., 2014). Importantly, these effects have been observed in both recall rates and in recognition accuracy. Of particular interest here is that animacy effects have been found in studies using the Remember/Know paradigm (Gardiner, 1988), in which participants indicate whether they specifically remember (R) contextual details of items they recognize (e.g., a feeling, a location), or whether they just know (K) that they have seen the items. Regarding animacy, it has been found that participants give more “R” responses to animate than inanimate items, whereas “K” responses do not differ reliably between the two types of item (Bonin et al., 2014; Bugaiska et al., 2016). This pattern of findings strongly suggests that animacy effects in memory are episodic in nature, in that episodic memory is characterized by the remembering of contextual information in (young) adults (e.g., Rawlinson and Kelley, 2021; Komar et al., 2022).

To the best of our knowledge, only one study has examined whether young children, like adults, show enhanced retention of animacy-related information (Aslan and John, 2016). In Aslan and John’s (2016) study, kindergarten and elementary school children (4–11 years) were tested. As in Nairne et al.’s (2013) study with adults, the children were presented with non-words paired either with properties characteristic of humans (e.g., speaks French) or animals (e.g., has fur), or with properties characteristic of inanimate things (e.g., has a lid). For each non-word (e.g., BULA, LAFE), children were asked to give a quick “living” or “non-living” response (forced choice), and after a retention interval of 3 min, they had to recognize the non-words. Non-words paired with human or animal characteristics were recognized better than those paired with inanimate properties; in other words, an “animacy effect” in memory was found in children. The advantage of animate over inanimate non-words was identical across age groups, suggesting developmental invariance of the benefit over the tested age range. The authors concluded that young children’s memory is tuned to process and retain information related to animacy. Hence, Aslan and John’s (2016) findings provide further support for the evolutionary view of memory put forward by Nairne et al. (2008). However, their study did not distinguish between recollection and familiarity. As proposed in the literature, retrieval using a recognition task relies on two distinct processes: recollection and familiarity. As stated above, adults have been shown to recognize animates better than inanimates (Bonin et al., 2014; Bugaiska et al., 2016; Rawlinson and Kelley, 2021; Komar et al., 2022), and importantly, the animacy effect has been observed on “Remember” but not on “Know” responses (Bonin et al., 2014). This pattern of findings supports the hypothesis that the animacy effect in memory is episodic in nature. Animacy enhances not only the quantity but also the quality of remembering; in other words, the effect is primarily driven by recollection. We believe that identifying the nature of animacy effects in young children is an important issue, which was not addressed by Aslan and John (2016). Therefore, the aims of the present study were to establish whether animacy effects are replicable in children, and more importantly, to determine whether these effects are episodic in nature, as found in adults (e.g., Bonin et al., 2014). The Remember/Know paradigm has rarely been used in studies with young children, but the available evidence suggests that the proportion of “Remember” responses made by young children (8–10 years) is smaller than that made by older children (11–13 and 14–16 years) and young adults (17–19 years); by contrast, there is no age-related difference in the proportion of “Know” responses (Billingsley et al., 2002). A recent study by Canada et al. (2022) found that other aspects of children’s cognitive development might enhance episodic memory performance, especially during middle childhood (e.g., 6–8 years; for a review see Schneider and Ornstein, 2019). This supports the view that middle childhood is a transitional period for the development of episodic memory and attention (Diaz et al., 2018).

To recap, the aim of this research was to study animacy effects in memory, and more specifically in recollection, in young children. Unlike Aslan and John’s (2016) study, which used non-words linked to animate and inanimate properties, we investigated the quality of retrieval of animate and inanimate words in order to investigate whether animacy effects emerge at a relatively young age, as found by Aslan and John (2016), and more importantly, whether these effects in children are episodic in nature.

## Materials and methods

### Participants

A total of 42 children from two age groups (younger and older elementary school children) took part in this study. One of the children in the younger group was excluded because her/his false alarm rates exceeded hit rates in all animate and inanimate conditions. The final sample thus comprised 20 younger elementary school children (6–7 years, *M* = 6.6 years), and 21 older elementary school children (10–12 years, *M* = 10.83 years). Prior to the study, we conducted a power analysis using G*Power (Faul et al., 2007) for sample size estimation based on the data of Aslan and John (2016). Their sample (*N* = 90) was divided into three age groups, but the authors did not provide data analyses for each age group because the animacy condition factor did not interact with age, *F*(4, 174) < 1. Our estimation is therefore based on the main effect of the animacy condition as a within-subject factor (3: human, animal and inanimate). The authors reported a main effect of this condition, *F*(2, 174) = 12.9, *MSE* = 0.03, *p* < 0.001, η = 0.129, with higher recognition of items related to humans (56.7%) and animals (57.4%) than inanimates (45.7%, *ps* < 0.001), while there was no difference between the two animate conditions (human vs. animal, (*p* = 0.769). The partial eta-squared effect size was η = 0.129. For a group assessed across three observations, with effect size specification as in GPower 3.0, an alpha of.05 and a power of.80, the minimum sample size needed with this effect size is *N* = 13. In the present experiment, we compared two animacy conditions (animate vs. inanimate) rather than three (human, animal and inanimate), as no difference was observed between the two animate categories in Aslan and John’s (2016) study. With this effect size, the minimum sample size for two repeated measures is *N* = 16. We rounded this figure up to *N* = 20 per age group, which is adequate to test the study hypothesis.

### Material and design

The participants performed a recognition memory test using the Remember/Know/Guess method. This study was carried out in the context of a research agreement (agreement no. 0482- 2021) between the laboratory, the university, the French national center for scientific research (CNRS) and the academic inspectorate (“Inspection Académique de Côte d’Or”). We conducted this study in accordance with the 1964 Declaration of Helsinki and we obtained written parental consent for each child. All participants were tested individually.

#### Stimuli for encoding

For the R/K/G paradigm, the material consisted of 24 nouns selected from the databases of Snodgrass and Vanderwart (1980) and Bonin et al. (2003). Each word referred to either an animate or an inanimate object.^1^ The words included 12 animate and 12 inanimate items, matched for surface variables (number of letters and bigram frequency), lexical variables (book frequency, subtitle frequency, age-of-acquisition, number of orthographic neighbors, and orthographic uniqueness point), and semantic variables (conceptual familiarity, imageability, image variability, concreteness, and emotional valence).^2^ Regarding age of acquisition, we selected words expected to be acquired by the children in our sample. The statistical characteristics of the controlled variables can be found in the Supplementary material. For the recognition task, we included twelve additional (“new”) words (6 animate and 6 inanimate), which matched the objective word frequency of the initial experimental words (“old”).

### Procedure

The children were tested individually, seated comfortably in a quiet room.

#### Encoding task

They were fitted with headphones so that they could hear the words perfectly. A word was presented every three seconds, and the children were asked to repeat each one out loud to ensure that they had heard it correctly. They were not instructed to learn the words, so encoding was incidental. Two lists were created, each with the same 24 words (12 animate and 12 animate) in a different order, so that half the children were presented with the words in one order and half with the words in the other order.

#### Distractor task

After the encoding task, the participants were given 2 min to perform the Cancelation subtest of the Wechsler Intelligence Scale for Children-Fifth Edition (WISC-V) (Wechsler, 2014). This test was used as an interference task.

#### Recognition task

They then performed the recognition task, in which all 36 words (24 targets and 6 animate and 6 inanimate fillers) were presented orally *via* headphones. The children had as much time as they wanted to respond. For each word, they were asked if they recognized it from the previously presented list. If so, they were instructed to give a remember (R), know (K), or guess (G) response: an R-response if they had a specific recollection of the learning sequence (e.g., it brought to mind a particular association, image, or some other personal experience, or because they recalled something about its appearance or position); a K-response if they were sure they recognized the word but had no conscious recollection of learning it); a G-response if they were not sure whether they had already seen the word or not. To ensure that the instructions were understood, they were asked the following question: *“Do you remember hearing this word before?”* If they answered yes, they were asked: *“Did you think of anything in particular when you heard this word, or did you think of nothing? For example, if you had heard the word bike, you might have thought of your bike, or of a cartoon with a bike in it, or a family bike ride.*” To ensure that the children had followed the instructions correctly, they were asked to explain two of their Remember and two of their Know judgments after the recognition phase.

#### Control task

Finally, the participants were given a naming test, in which they had to name pictures corresponding to the words they had heard during the encoding phase. This task was included in addition to the age-of-acquisition control variable to ensure that all the children knew the words shown at encoding. All the children correctly named the pictures.

## Results

### Analysis of Remember/Know paradigm

**FIGURE 1:**
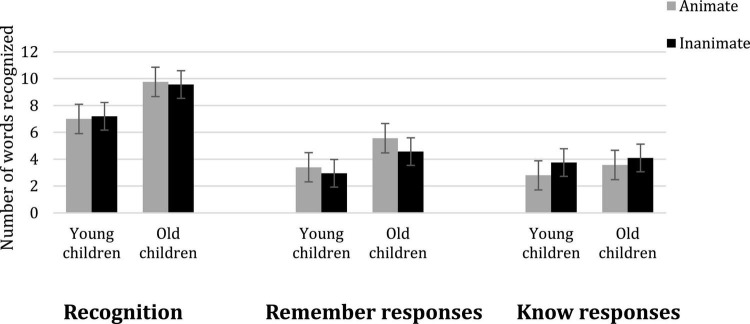
Mean number of correctly recognized words (Hits-FA) as a function of age (young vs. older children) and type of words (animate vs. inanimate) for recognition, remember responses and know responses. Error bars represent standard errors of the mean.

The hits minus false alarms and standard errors for overall recognition, Remember and Know responses are presented in Figure 1.^3^

To test the effect of animacy and age on overall recognition, Remember responses and Know responses, we conducted a 2 × 2 ANOVA with Animacy as a within-subject factor and Age as a between-subjects factor on these measures.

#### Overall recognition

The younger children recognized fewer words from the previously presented list than the older children, *F*(1,39) = 25.69, *p* < 0.001, η^2^_*p*_ = 0.40. The main effect of Type of words was not significant, *F*(1,39) < 0.001, *p* = 0.99. Finally, as shown in Figure 1, the interaction between Age and Type of words was not significant, *F*(1,39) = 0.33, *p* = 0.57.

#### Remember responses

The ANOVA of R-responses revealed a reliable main effect of Age, *F*(1,39) = 6.10, *p* = 0.02, η^2^_*p*_ = 0.14, the younger children recollecting fewer words than the older children. A main effect of Type of words emerged, *F*(1,39) = 5.72, *p* = 0.02, η^2^_*p*_ = 0.13, showing that animate words gave rise to more recollective experience than inanimate words. Finally, the interaction between Age and Type of words was not significant, *F*(1,39) = 0.82, *p* = 0.37. Nevertheless, as shown in Figure 1, the animacy effect was greater in older than in younger children. A Paired sample *t*-test were conducted to determine the effect of animacy on the remember responses in the older and younger children. Results showed that older children recollected animate words better than inanimate words, *t*(20) = 2.14, *p* = 0.0451 (*M* = 5.57 and *M* = 4.57). However, the difference between animate and inanimate words for younger children was not significant, *t*(19) = 1.18, *p* = 0.25 (*M* = 3.4 and *M* = 2.95).

#### Know responses

The analysis of K-responses (Figure 1) revealed that there was no reliable effect of age, *F*(1,39) = 0.72, *p* = 0.40. The main effect of Type of words was significant, *F*(1,39) = 5.7, *p* = 0.02, η^2^_p_ = 0.13, with more Know responses for inanimate than animate words. Finally, there was no reliable interaction between Age and Type of words, *F*(1,39) = 0.48, *p* = 0.49.

## Discussion

Previous studies have established that animacy effects in memory are found on R-responses (an index of recollection) but not on K-responses (Bonin et al., 2014; Bugaiska et al., 2016; Rawlinson and Kelley, 2021; Komar et al., 2022), suggesting that the animacy effect is episodic in nature. However, these studies only involved young adults. We believe that it is worth investigating whether this is also the case for children. To the best of our knowledge, this is the first study to investigate the role of animacy in children’s recollection, and to this end we asked a simple question: Do younger and older children remember animates better than inanimates, in the same way as young adults? Using a remember-know procedure with a sample of children aged 6–12 years, the present findings do not provide a clear-cut answer to this question, some conclusions varying with age group. First of all, there was no reliable animacy effect on overall recognition for either age group. However, when the recognition performance of older children was divided between “recollection” and “familiarity,” we found an animacy effect on recollection but not on know responses. This suggests that the animacy effect in older children is due to an increase in recollection. Importantly, our findings are in line with those of Aslan and John (2016), but extend them by suggesting that the animacy effect is underpinned by episodic memory processes from the age of 10–11 years. It is particularly noteworthy that recollection processes are involved for animate but not inanimate words from an early age. It seems that inanimate words are not encoded with contextual details, and therefore this type of information is not helpful when they have to be remembered. Finally, our findings in older children are in line with the literature on young adults (Bonin et al., 2014; Bugaiska et al., 2016; Rawlinson and Kelley, 2021; Komar et al., 2022), showing that the animacy effect in memory is related to the quality of remembering, but has no effect on knowing. Overall, the current findings are consistent with a functional-evolutionary view of human memory, which posits that our memory systems have been tuned by natural selection due to pressures faced by our hunter-gatherer ancestors in the distant past. In particular, it has been suggested that recurrent interactions with animates exerted strong evolutionary pressure on humans, leading to the development of memory systems that prioritize the processing and remembering of animates.

Regarding the younger children, there was no reliable animacy effect for either overall recognition or recollection. This is at odds with the results of Aslan and John (2016), who found that the benefit of animate non-words was identical across age groups, suggesting developmental invariance of the benefit over the age range tested (i.e., 4–11 years). They concluded that young children’s memory is “tuned” to process and retain animacy from a very early age (4 years). While we cannot provide a satisfactory explanation for this discrepancy between our findings and theirs, we suggest that it could be linked to the way the stimuli were presented. In Aslan and John’s (2016) study, the children were asked to respond rapidly about the animacy status of non-words (e.g., BULA, LAFE) based on properties that referred to humans (e.g., speaks French) or animals (e.g., has fur) or to inanimate entities (e.g., has a lid). In our study, we did not ask the children to pay attention at encoding to the status (animate versus non-animate) or to certain semantic characteristics of the words, but simply to read them aloud. It is possible that this difference in the protocol was significant, because information about animacy had to be inferred from reading the words. As previously suggested, imagery skills could explain the animacy effect on memory performance (Blunt and VanArsdall, 2021). Immature imagery skills could therefore explain the lack of animacy effect in young children. The ability of children to form internal representations including movements is indeed still a matter of debate. From a Piagetian perspective, mental representations develop with age and are constrained by the characteristics of the stages of cognitive development. According to Piaget and Inhelder (1966), children under the age of 7 to 8 years are not able to represent movements, limiting mental representations to static states. The concrete operational stage would provide the framework within which transformations or movements can be represented. However, conflicting results suggest that 4- to 5-year-old children use kinetic imagery to solve mental rotation tasks (e.g., Marmor, 1972). A major difference between Marmor’s study and those of Piaget and Inhelder is that the children were instructed to use kinetic imagery to solve the rotation task in the former but not in the latter. If young children do not perceive the relevance of using kinetic imagery during a mental rotation task, it is very likely that they will not spontaneously represent animates in motion when nothing invites them to do so, as in the present study, while they are able to form kinetic imagery of animates when the procedure draws attention to their “animated” characteristics, as in Aslan and John’s (2016) study. Further studies should address this issue by contrasting the effect of these two procedures on the emergence of the animacy effect in young children.

Turning to recollection, the difference in the effect of animacy between young and older children is not totally unexpected. As mentioned in the Introduction, it has been suggested that other aspects of children’s cognitive development may enhance episodic memory performance, notably during middle childhood (e.g., 7 years; for review see Schneider and Ornstein, 2019). A study conducted with 6- to 8-year-old children suggested that middle childhood is a transitional period for the development of episodic memory and attention (Diaz et al., 2018). Similarly, it is likely that environmental changes interact with the development of multiple cognitive processes and contribute to improvements during childhood.

To examine further the animacy effect in episodic memory in very young children, it would be interesting to repeat this experiment, but asking the children to read the words and say whether they refer to animate or inanimate entities. It would also be interesting to replicate Aslan and John’s (2016) study with non-words by adding a recognition task using the Remember/Know paradigm to examine the extent to which animacy effects in young children rely on recollection. It is possible that the animacy effect in episodic memory does not emerge till a later age and that it is related to the development of episodic memory. In that case, animacy effects could be used as an index of episodic memory functioning/maturation in children.

To conclude, do children, like young adults, remember animates better than inanimates? The answer is “yes” for older children, for whom the animacy effect relies on recollection. As found with young adults, the animacy effect in memory in older children (10–12 years, *M* = 10.83 years) is episodic in nature. For younger children, the tentative answer is “no,” but further studies are clearly needed to gain a better understanding of when (and how) this memory effect emerges in young children.

## Data availability statement

The raw data supporting the conclusions of this article will be made available by the authors, without undue reservation.

## Ethics statement

The studies involving human participants were reviewed and approved by the research agreement (agreement n°: 0482- 2021) between the laboratory, the university, the French National Center for Scientific Research (CNRS) and the academic inspectorate (“Inspection Académique de Côte- d’Or”). Written informed consent to participate in this study was provided by the participants’ legal guardian/next of kin.

## Author contributions

AB, AW, and PB contributed to the conception and design of the study and performed the statistical analysis, and contributed to the manuscript writing. AB and AW performed the investigation. AB wrote the first draft of the manuscript. All authors approved the submitted version.

## Footnotes

1 We used a broad definition of animacy as in previous literature on animacy effects in memory (e.g., Gelin et al., 2019). Thus, we considered animate words to refer to living things that can move on their own (e.g., a cow) and inanimate words to refer to non-living things that cannot move on their own (e.g., a car). The authors used this definition to code the words as animates versus inanimates; full agreement for all the words was reached. Lists of words can be found in the Supplementary material.

2 We are aware that the norms listed here are based on adults. Unfortunately, norms on these dimensions are not available for children in French.

3 A table summarizing the proportions of correct and false alarm responses for general recognition, R-responses and K-responses as a function of age and word type can be found in Table 2 in the Supplementary material.
