# Supplementary material for: Do young children, like young adults, remember animates better than inanimates?
Source: Front Psychol. 2023 May 10;14:1141540. doi: 10.3389/fpsyg.2023.1141540 (PMC10206057; doi:10.3389/fpsyg.2023.1141540)
Supplement: Supplementary file 1 [file Table_1.docx]

**Supplementary material**

*Table 1. Statistical characteristics (means, standard deviations, range: min-max, t-tests of the means) of the control variables for animate and inanimate stimuli*

|  | animate | | | inanimate | | |  |
| --- | --- | --- | --- | --- | --- | --- | --- |
|  | Mean | SD | Range | Mean | SD | Range | t-test |
| Number of letters* | 6.91 | 1.75 | 4-10 | 6.58 | 1.93 | 4-12 | *t* < 1 |
| Bigram frequency (per million words)* | 9324.5 | 2140.74 | 5841-12807 | 8294 | 2312.09 | 5151-12350 | *t* < 1 |
| Book frequency* | 21.02 | 30.83 | 1.35-110.92 | 28.62 | 22.95 | 1.62-74.59 | *t* < 1 |
| Subtitle frequency* | 19.48 | 24.72 | 0.69-85.42 | 18.71 | 14.12 | 0.77-48.61 | *t* < 1 |
| Age-of-acquisition (1-5)** | 2.01 | 0.41 | 1.35-2.8 | 2.28 | 0.44 | 1.62-3 | *t* < 1 |
| Number of orthographic neighbors* | 3.08 | 4.29 | 0-14 | 1.08 | 1.38 | 0-5 | *t* < 1 |
| Orthographic uniqueness point* | 5.58 | 2.14 | 0-9 | 6 | 1.47 | 0-9 | t < 1 |
| Conceptual familiarity** | 2.68 | 0.74 | 1.9-4.55 | 3.01 | 1.01 | 1.53-4.7 | *t* < 1 |
| Imageability*** | 4.59 | 0.25 | 4.12-4.96 | 4.49 | 0.23 | 4.08-4.84 | *t* < 1 |
| Image variability** | 2.92 | 0.62 | 1.9-4.17 | 2.82 | 0.67 | 1.6-3.8 | *t* < 1 |
| Concreteness*** | 4.63 | 0.36 | 3.69-4.91 | 4.75 | 0.23 | 4.14-5 | t < 1 |
| Emotional valence*** | 3.40 | 0.86 | 1.25-4.44 | 3.46 | 0.49 | 2.48-4.32 | *t* < 1 |

*Notes.* *values taken from Lexique (<http://www.lexique.org/>**;** New, B., Pallier, C., Brysbaert, M., Ferrand, L. (2004)); **All the scales are rated on 5 points. The values were obtained from Bonin et al. (2013); *** All the scales are rated on 5 points. The values were obtained from **Bonin,** Méot, Aubert, Malardier, Niedenthal, & Capelle-Toczek, 2003).

**Animate**

French English

Pigeon Pigeon

Moustique Mosquito

Marin Sailor

Cheval Horse

Chat Cat

Canard Duck

Poule Chicken

Coccinelle Ladybug

Papillon Butterfly

Danseuse dancer

Magicien Magician

Ecureuil Squirrel

**Inanimate**

French English

Bibliothèque Bookcase

Coffre Trunk

Brouette Wheelbarrow

Echelle Ladder

Flûte Flute

Chapeau hat

Violon Violin

Chemise Shirt

Râteau Rake

Canapé Sofa

Piano Piano

Jupe Skirt

*Table 2*: *Proportions of hits and false alarms and standard deviations for overall recognition, remember and know responses as a function of age (young vs. older children) and type of words (animate vs. inanimate).*

|  | Younger children  (n=20) | | | | Older children  (n=21) | | | |
| --- | --- | --- | --- | --- | --- | --- | --- | --- |
|  | animate | | inanimate | | animate | | inanimate | |
|  | M (SD) | | M (SD) | | M (SD) | | M (SD) | |
| *Hits* |  |  |  |  |  |  |  |  |
| Overall recognition | .33 (.09) | | .32 (.08) | | .42 (.07) | | .42 (.08) | |
| Remember | .14 (.13) | | .13 (.09) | | .23 (.09) | | .19 (.11) | |
| Know | .13 (.09) | | .16 (.11) | | .15 (.10) | | .17 (.09) | |
| Guess | .06 (.07) | | .03 (.07) | | .04 (.05) | | .06 (.06) | |
| *False alarms* |  | |  | |  | |  | |
| Overall recognition | .07 (.10) | | .04 (.07) | | .03 (.06) | | .05 (.06) | |
| Remember | .00 (.00) | | .01(.03) | | .00 (.00) | | .00 (.02) | |
| Know | .02 (.05) | | .01 (.02) | | .01 (.03) | | .01 (.03) | |
| Guess | .05 (.09) | | .02 (.05). | | .02 (.05) | | .04 (.06) | |
